# New Sequence Variants in HLA Class II/III Region Associated with Susceptibility to Knee Osteoarthritis Identified by Genome-Wide Association Study

**DOI:** 10.1371/journal.pone.0009723

**Published:** 2010-03-18

**Authors:** Masahiro Nakajima, Atsushi Takahashi, Ikuyo Kou, Cristina Rodriguez-Fontenla, Juan J. Gomez-Reino, Tatsuya Furuichi, Jin Dai, Akihiro Sudo, Atsumasa Uchida, Naoshi Fukui, Michiaki Kubo, Naoyuki Kamatani, Tatsuhiko Tsunoda, Konstantinos N. Malizos, Aspasia Tsezou, Antonio Gonzalez, Yusuke Nakamura, Shiro Ikegawa

**Affiliations:** 1 Laboratory for Bone and Joint Diseases, Center for Genomic Medicine, RIKEN, Tokyo, Japan; 2 Laboratory for Statistical Analysis, Center for Genomic Medicine, RIKEN, Tokyo, Japan; 3 Laboratorio Investigacion 10 and Rheumatology Unit, Hospital Clinico Universitario de Santiago, Santiago de Compostela, Spain; 4 Department of Medicine, University of Santiago de Compostela, Santiago de Compostela, Spain; 5 The Center of Diagnosis and Treatment for Joint Disease, Drum Tower Hospital Affiliated to Medical School of Nanjing University, Nanjing, China; 6 Department of Orthopaedic Surgery, Mie University Faculty of Medicine, Mie, Japan; 7 Department of Pathomechanisms, Clinical Research Center for Rheumatology and Allergy, National Hospital Organization Sagamihara National Hospital, Kanagawa, Japan; 8 Laboratory for Genotyping Development, Center for Genomic Medicine, RIKEN, Kanagawa, Japan; 9 Laboratory for Medical Informatics, Center for Genomic Medicine, RIKEN, Kanagawa, Japan; 10 Department of Orthopaedics University of Thessaly, Larissa, Greece; 11 Institute for Biomedical Research and Technology, Larissa, Greece; 12 Department of Biology, University of Thessaly Medical School, Larissa, Greece; 13 Human Genome Center, Institute of Medical Science, The University of Tokyo, Tokyo, Japan; 14 Center for Genomic Medicine, RIKEN, Kanagawa, Japan; Ohio State University, United States of America

## Abstract

Osteoarthritis (OA) is a common disease that has a definite genetic component. Only a few OA susceptibility genes that have definite functional evidence and replication of association have been reported, however. Through a genome-wide association study and a replication using a total of ∼4,800 Japanese subjects, we identified two single nucleotide polymorphisms (SNPs) (rs7775228 and rs10947262) associated with susceptibility to knee OA. The two SNPs were in a region containing HLA class II/III genes and their association reached genome-wide significance (combined *P* = 2.43×10^−8^ for rs7775228 and 6.73×10^−8^ for rs10947262). Our results suggest that immunologic mechanism is implicated in the etiology of OA.

## Introduction

We are living in the “Bone and Joint Decade” (http://www.boneandjointdecade.org/). As the WHO initiative shows, bone and joint diseases are serious problems all over the world, putting us under severe medical, economical and social burden. Osteoarthritis (OA; MIM 165720) is one of the most common diseases among them. OA affects synovial joints of all over the body, mainly knee, hip, hand and spine. OA is characterized by progressive loss of articular cartilage and, often, proliferation of synovium and bone, which lead to pain, loss of joint function and disability. More than tens of millions patients in the world are suffering from this non-lethal, but intractable disease, and the number is relentlessly increasing; however, its etiological picture remains unclear and we have no fundamental treatment for it.

OA is a polygenic disease. Both environmental and genetic factors contribute to its etiology and pathogenesis [1]. To understand its genetic factor, identification of its susceptibility gene(s) must be the first step. Many OA susceptibility genes identified by candidate-gene association studies have been reported, but only a few have supporting functional evidence and replication of the results in different populations [1], [2]. Large-scale association studies including the genome-wide association study (GWAS) using high-density single nucleotide polymorphisms (SNPs) have been reported by a few groups in Asia and Europe [3]–[6], but only a gene fulfilled genome-wide significance level [2]. The genetic basis of OA susceptibility remains largely uncharacterized. To identify OA susceptibility gene(s), we conducted a GWAS for knee OA and identified two SNPs with genome-wide significance level.

## Methods

### Samples

Characteristics of each cohort group are shown in Table 1. Case samples of GWAS for the Japanese population were obtained from several medical institutes in Japan, as previously described [5], [7]. Knee OA was diagnosed on the basis of clinical and radiographic findings using previously described criteria [5], [7]. Rheumatoid arthritis (RA) and polyarthritis associated with autoimmune diseases were excluded, as were secondary OA due to crystal deposition (gout and pseudogout), posttraumatic OA and infection-induced OA. Patients who had clinical and radiographic findings suggestive of skeletal dysplasias, including overt short stature, multiple symmetric involvements of epiphyses and a definitely positive Mendelian family history were also excluded from the study. The control groups consisted of 3,396 individuals that were registered in the Leading Project for Personalized Medicine in the Ministry of Education, Culture, Sports, Science and Technology, Japan as the subjects with diseases unrelated to OA and the volunteers in the Osaka-Midosuji Rotary Club, Osaka, Japan [8]. For replication study, we recruited population-based cohorts from inhabitants of Odai and Minami-ise town (previously Miyagawa village and Nansei town, respectively in the Mie prefecture in Japan) [9]. The Spanish and Greek knee OA and control populations were recruited as described previously from the Hospital Clinico de Santiago, the Departments of Biology and Genetics and of Orthopaedics, University of Thessaly and the Institute of Musculoskeletal Sciences [10]. All the participants provided written informed consent. This research project was approved by the ethical committees at Center for Genomic Medicine (formerly, SNP Research Center), RIKEN and the participating institutions.

**Table 1:** Basal characteristics of the subjects.

Cohort		Source	Platform	Number of samples	Nationality	Female (%)	Age (mean +/− sd)	BMI (mean +/− sd)	Severity^a^ (% severe OA)
**GWAS**									
	knee OA	RIKEN	Illumina HumanHap550	899	Japanese	759 (84.4)	71.6+/−7.6	24.9+/−3.6	76.5
	control	ORC+BioBank Japan	Illumina HumanHap550	3,396	Japanese	1,491 (43.9)	52.5+/−15.2	22.5+/−3.7	–
**Replication**									
Japanese									
	knee OA	RIKEN	Invader assay	167	Japanese	124 (74.3)	73.8+/−6.1	24.5+/−3.3	48.5
	control	RIKEN	Invader assay	347	Japanese	223 (64.3)	65.9+/−8.7	22.3+/−2.7	–
European Caucasian									
	knee OA	Santiago de Compostela	SNaPshot	243	Spanish	197 (81.1)	68.0+/−5.7	32.8+/−4.8	ND^b^
	control	Santiago de Compostela	SNaPshot	426	Spanish	165 (38.7)	68.4+/−9.1	28.3+/−3.8	–
	knee OA	University of Thessaly	SNaPshot	570	Greek	468 (82.1)	65.8+/−8.7	29.1+/−3.3	77.1
	control	University of Thessaly	SNaPshot	645	Greek	417 (64.6)	59.5+/−11.6	25.4+/−3.7	–

OA: osteoarthritis, ORC: Osaka-Midosuji Rotary Club.

a Kellegren-Laurence grade ≥3 was considered as severe OA.

b All cases underwent TKR (total knee replacement) surgery.

### SNP genotyping

For the GWAS, we genotyped 906 patients with OA and 3,396 controls using Illumina HumanHap550v3 Genotyping BeadChip. After excluding seven cases with call rate of <0.98, we applied SNP QC (call rate of ≥0.99 in both cases and controls and *P* value of Hardy-Weinberg equilibrium test of ≥1.0×10^−6^ in controls). Finally, 459,393 SNPs on autosomal chromosomes passed the QC filters and were further analyzed. Among the SNPs analyzed in the GWAS, we selected top 15 SNPs showing the smallest *P* values (*P*<1×10^−5^) for the replication study using an independent 514 Japanese subjects from a resident cohort. SNPs with minor allele frequency of ≤0.1 in both case and control samples were excluded from the further analysis. In the replication analysis, we genotyped SNPs using the multiplex PCR-based invader assay (Third Wave Technologies) or by direct sequencing of PCR products using ABI 3700 DNA analyzers (Applied Biosystems), or by SNaPshot Multiplex System (Applied Biosystems) according to manufacturers' protocols.

### Statistical analysis

In the GWAS and replication analyses, we applied Fisher's exact test to two-by-two contingency table in three genetic models: an allele frequency model, a dominant-effect model, and a recessive-effect model. We conducted the meta-analysis using the Mantel-Haenszel method. We examined heterogeneity among studies by using the Breslow-Day test. Significance levels after the Bonferroni correction for multiple testing were *P* = 1.09×10^−7^ (0.05/459,393). Age, gender- and BMI-adjusted odds ratios were obtained by logistic regression analysis [11]. Odds ratios and confidence intervals were calculated using the risk allele as a reference. We analyzed the haplotype association using Haploview software [12]. We conducted a principal component analysis to detect population stratification [13].

### Software

For general statistical analysis, we used R statistical environment version 2.6.1 or Microsoft Excel. Drawing the LD map, estimation of haplotype frequencies and analysis of haplotype association were performed by Haploview software.

## Results

**Figure 1:**
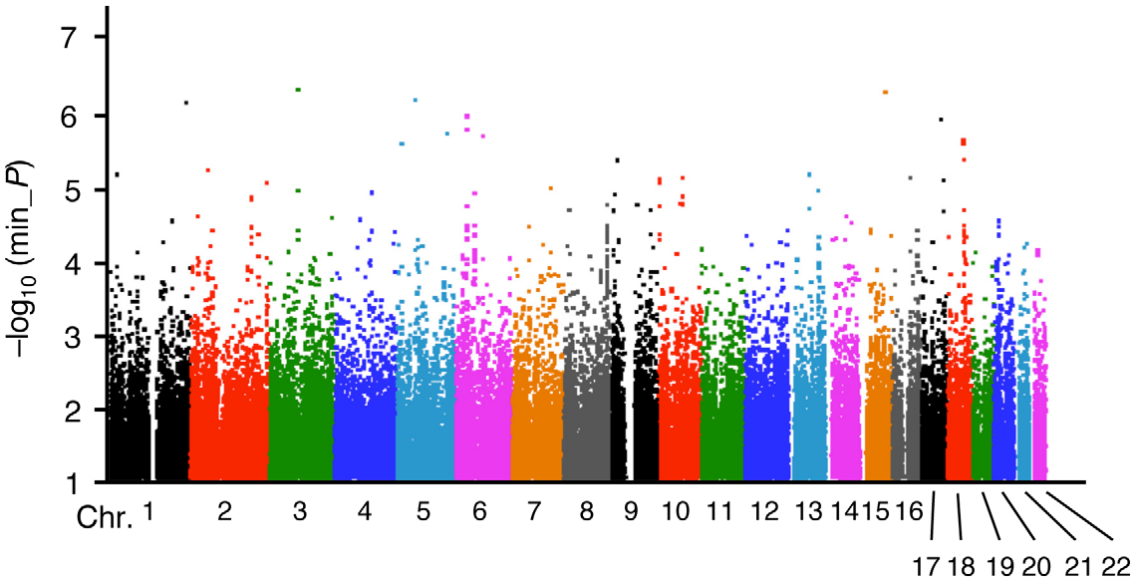
Results of a genome-wide association study (–log_10_ *P* value plot). Each *P* value is the minimum of Fisher's exact tests for three models: dominant, recessive and allele frequency model.

**Figure 2:**
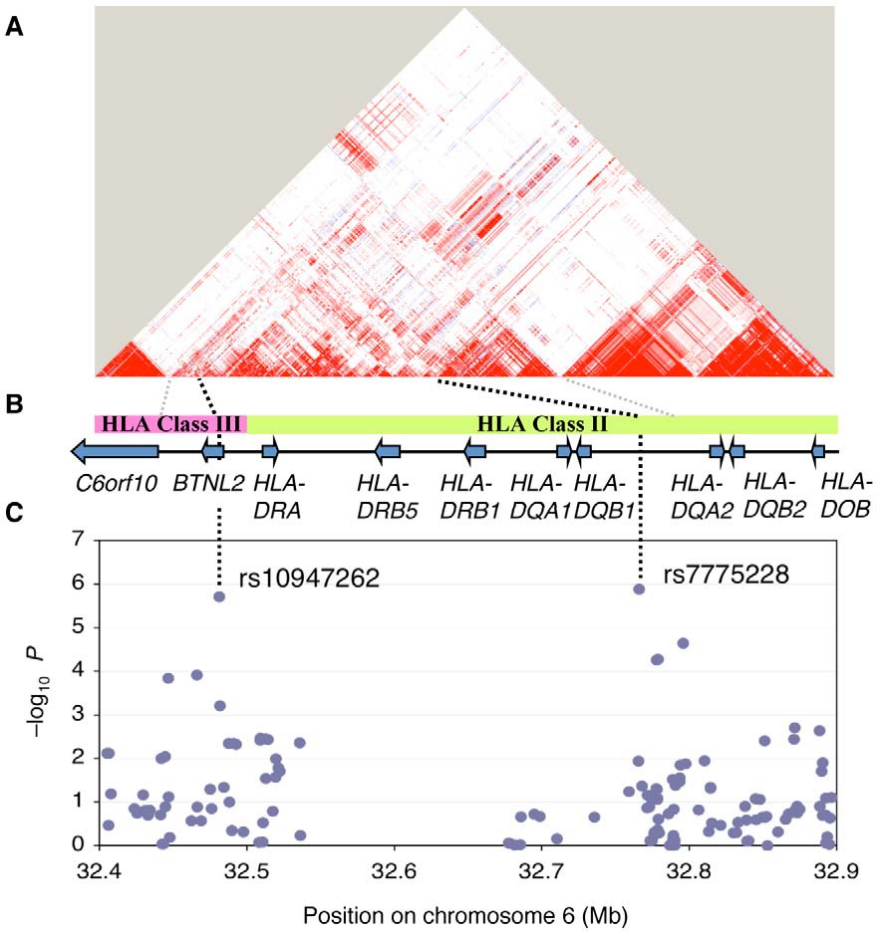
Case-control association analysis and linkage disequilibrium (LD) map of the HLA class II/III region of chromosome 6. (A) The LD map based on *D*' was drawn using HapMap data release 24 for the JPT population. (B) Genomic structure within the extended HLA-II/III region. (C) Results of GWAS for osteoarthritis in Japanese population. The log_10_-transformed *P* values are plotted on the *y* axis.

To identify genetic variants that determine OA susceptibility, we conducted a GWAS in Japanese knee OA. We examined 906 individuals with knee OA and 3,396 control individuals (Table 1) using Illumina HumanHap550v3 Genotyping BeadChip. After confirming the data quality, we compared the results of 459,393 SNPs between cases and controls by Fisher's exact test for three genetic models: allelic, dominant or recessive (Figure 1). Fifteen SNPs selected for the replication study that had the smallest *P* values (minimum *P*<1×10^−5^) were next genotyped in an independent set of 167 Japanese knee OA individuals and 347 Japanese controls from a resident cohort study. Through these studies, only two SNPs, rs7775228 (combined *P* = 2.43×10^−8^; OR = 1.34; 95% CI = 1.21–1.49) and rs10947262 (combined *P* = 6.73×10^−8^; OR = 1.32; 95% CI = 1.19–1.46) were significant even after the Bonferroni correction for multiple testing (*P* = 1.09×10^−7^) (Table 2). The two SNPs showing significant associations are located within a 340-kb region within the HLA locus, including *BTNL2*, *HLA-DRA*, *HLA-DRB5*, *HLA-DRB1*, *HLA-DQA1* and *HLA-DQB1* (Figure 2). Although the HLA region is known to show extensive linkage disequilibrium (LD) spanning over 7 Mb, only SNPs in the 340-kb region showed strong associations with OA (Figure 2), and SNPs outside of this region did not have significant association.

**Table 2:** Association of rs7775228 and rs10947262 with knee osteoarthritis.

SNP (Nearest gene)	Allele	Population	Minor allele Frequency				
			Case	Control	OR (95% CI)^a^	*P* b	*P*_het_ c
rs7775228	C/T	Japanese					
(*HLA-DQB1*)		GWAS	0.318	0.379	1.31 (1.18–1.47)	1.38 E-06	
		Replication	0.290	0.385	1.53 (1.15–2.02)	3.07 E-03	
		Combined^d^			1.34 (1.21–1.49)	2.43 E-08	0.33
		European Caucasian					
		Spanish	0.194	0.209	1.10 (0.83–1.45)	0.521	
		Greek	0.094	0.075	0.78 (0.58–1.03)	0.084	
		European combined^e^			0.93 (0.76–1.13)	0.178	0.09
		All combined^f^			–	–	0.003
rs10947262	C/T	Japanese					
(*BTNL2*)		GWAS	0.358	0.419	1.30 (1.16–1.44)	2.45 E-06	
		Replication	0.332	0.422	1.47 (1.12–1.93)	5.74 E-03	
		Combined^d^			1.32 (1.19–1.46)	6.73 E-08	0.40
		European Caucasian					
		Spanish	0.122	0.136	1.13 (0.81–1.59)	0.465	
		Greek	0.068	0.094	1.43 (1.06–1.92)	0.019	
		European combined^e^			1.29 (1.03–1.61)	0.025	0.32
		All combined^f^			1.31 (1.20–1.44)	5.10 E-09	0.63

Odds ratios (ORs) and *P* values for the independence test were calculated by the Mantel-Haenszel method.

a OR of the risk allele from the two-by-two allele frequency table.

b P values of the Pearson's χ^2^ test for the allele model.

c Results of the Breslow-Day test.

d Meta-analysis of Japanese studies.

e Meta-analysis of European studies.

f Meta-analysis of all studies.

**Figure 3:**
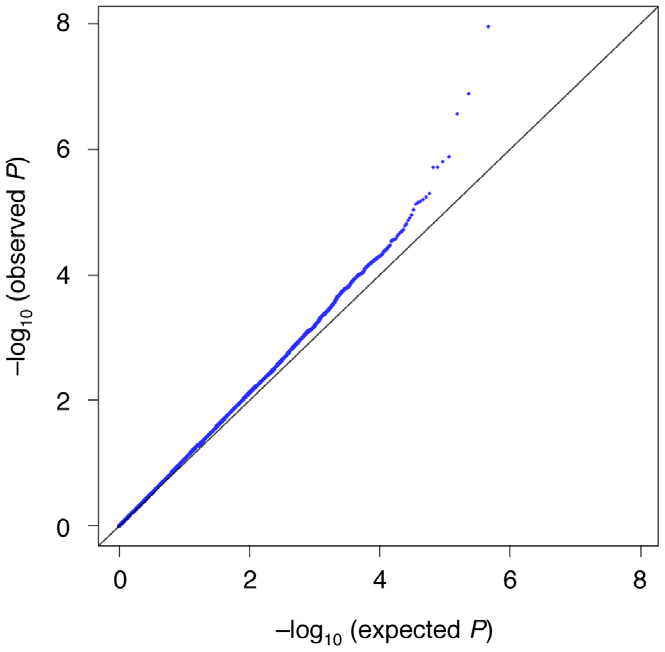
GWAS of knee osteoarthritis. Q-Q plot with Cochrane-Armitage trend *P* in the GWAS**.** Horizontal and vertical lines represent expected *P* values under a null distribution and observed *P* values, respectively. The genetic inflation factor lambda is 1.08.

Application of the Cochrane-Armitage test to all the tested SNPs indicated that the genetic inflation factor lambda was 1.08 for GWAS (Figure 3), implying a low possibility of false positive associations due to population stratification. We also carried out age, gender- and BMI-adjusted analysis using a logistic regression model, and confirmed similar association after adjustment (data not shown). The principal component analysis [13] revealed that there was no evidence for population stratification between the two control groups used for the GWAS (Figure S1).

To check the association of rs7775228 and rs10947262 in different ethnic populations, we examined the association of the SNPs with knee OA in two European Caucasian populations from Greece and Spain. We genotyped a total of 813 OA and 1,071 control subjects (Table 1). We conducted the meta-analysis using the Mantel-Haenszel method. The combined European results for rs7775228 were not significant with OR (95%CI) of 0.93 (0.76–1.13) (Table 2), while those for rs10947262 were supportive with OR (95%CI) of 1.29 (1.03–1.61). rs10947262 showed replication in the Greek population and the same trend in the Spanish population (Table 2). A meta-analysis of the Japanese and two European studies gave more significant association (combined *P* = 5.10×10^−9^).

We estimated the pairwise LD indexes (*D*' and *r^2^*) between rs7775228 and rs10947262 using the genotype data of Japanese populations (GWAS and the replication study), and found that they were in strong LD with each other (*D*' = 0.82, *r^2^* = 0.56). They formed two frequent haplotypes (Haplotype I and II; Table 3) accounting for about 90% of all observed chromosomes. The haplotypes were also significantly associated with knee OA; Haplotype I, the most frequent haplotype was a risk haplotype (*P* = 1.48×10^−8^; OR = 1.33; 95% CI = 1.20–1.46).

**Table 3:** Haplotype association analysis for knee osteoarthritis susceptibility SNPs, rs7775228 and rs10947262.

		Frequency		Haplotype effect^b^	
Haplotype^a^		Case	Control	OR (95% CI)	P value
I	TC	0.610	0.541	1.33 (1.20–1.46)	1.48 E-8
II	CT	0.277	0.340	0.74 (0.67–0.83)	5.07 E-8
III	CC	0.077	0.080	0.96 (0.80–1.15)	0.653
IV	TT	0.037	0.040	0.91 (0.71–1.18)	0.475

OR: odds ratio, CI: confidence interval.

a Alleles of rs7775228 (C/T) and rs10947262 (C/T) with this order.

b A haplotype vs. all others.

## Discussion

We performed a GWAS followed by a replication in an independent population using a total of ∼4,800 Japanese subjects, and identified two SNPs (rs7775228 and rs10947262) in the HLA class II/III locus associated with susceptibility to knee OA. To our knowledge, this study represents the first GWAS of OA with extensive coverage (∼550,000 markers) and definite genome-wide significance even after Bonferroni's correction, which is very conservative. There were no effects of population stratification and confounding factors. Since two groups of controls were used in the GWAS, we evaluated the possibility of genetic heterogeneity between the two groups by a principal component analysis and found it unlikely (Figure S1). Although there was large age difference between the case and control groups of GWAS (Table 1), significant association was observed after the age adjustment.

In the NCBI genome database, rs7775228 and rs10947262 located between upstream region of *HLA-DQA2* and *HLA-DQB1*, and within the intron 1 of *BTNL2*, respectively (Figure 2). *HLA-DQA2* and *HLA-DQB1* encode HLA-DQ α and β chains, which belong to the HLA class II molecules. HLA class II molecules are expressed in antigen presenting cells (B lymphocytes, dendritic cells and macrophages) and play a central role in the immune system by presenting peptides derived from extracellular proteins [14]. The HLA-DQA*2* protein is expressed, but at a very low level in comparison with the HLA-DQA1 protein [15], [16]. Moreover, the HLA-DQA2 α chain does not dimerize with class II β chains [16]. *BTNL2* encodes butyrophilin-like 2, a member of butyrophilin family that shares sequence homology with the B7 co-stimulatory molecules. BTNL2 regulates T-cell activation through unknown receptor, distinct from CD28 and CTLA-4 [17]. In Japanese population, the haplotype association was more significant than those of respective SNPs (Tables 2 and 3). Therefore, there may be hidden SNP(s) with a lower *P* value than rs7775228 and rs10947262, or the haplotype may be implicated in the OA susceptibility. An association of sarcoidosis with rs2076530, a coding SNP on exon 5 of the *BTNL2* gene has been reported [18], but the SNP was not in LD with rs10947262 (*D*' = 0.11, *r^2^* = 0).

The 340-kb region of HLA locus, where the two SNPs are located also includes *HLA-DRA*, *HLA-DRB1*, *HLA-DRB3*, *HLA-DRB4*, *HLA-DRB5* and *HLA-DQA1*. *HLA-DRA*, *HLA-DRB1/3/4/5* and *HLA-DQA1* encode HLA-DR α, β and HLA-DQ α chains, which could also belong to the HLA class II molecules. *HLA-DRB1* is present in all individuals. Allelic variants of *HLA-DRB1* are linked with either none or one of the genes *HLA-DRB3*, *HLA-DRB4* and *HLA-DRB5* [19]. Among these genes, *HLA-DRB1* is strongly associated with RA. Some subtypes of HLA-DRB1 alleles, such as *0101, *0401, and *0405, is associated with RA [20], but not with generalized OA [21].

Although OA has generally been considered a non-inflammatory disease, accumulating evidences suggest that this is not the case. Inflammation involving activated T cells in the synovial membrane of OA patients is well documented [22]. Recently, we identified a genetic variant of *EDG2* gene encoding lysophosphatidic acid receptor associated with knee OA [23]. A GWAS has identified a genetic variant of the *PTGS2* gene encoding cyclooxygenase-2 involved in risk for knee OA [6]. These genetic associations of genes such as *EDG2* and *PTGS2* underscore the potential role of inflammatory pathways in the pathogenesis of knee OA.

Several studies have suggested associations of OA with HLA class I and class II alleles. Study on generalized OA revealed association with HLA A1-B8 in Caucasian [24] and with HLA-Cw4 in Japanese [21]. An association of the HLA-DRB1*02 alleles with knee and hip OA was identified in a cohort of 106 patients [25]. Interestingly, chondrocyte, which are normally HLA-DP, DQ and DR-negative, become positive for them in OA [26], [27], suggesting their function as antigen-presenting cells. Cartilage fragments are mechanically shaved from the joint surface and frequently found in the synovial membrane of OA patients [28]. So, physical interaction between chondrocytes and T cells is conceivable. Peripheral blood T cells from OA patients show significantly higher proliferative responses to autologous chondrocytes [27]. Our results further support the concept that OA is an immunologic disorder.

## Supporting Information

Figure S1 Principal component analysis of GWAS samples. Samples in the GWAS and in HapMap database are analyzed by a program of Smartpca [12], and plotted for the first (X axis) and the second (Y axis) principal component (PC), respectively.
(0.16 MB TIF)
