# Supplementary figures and images for: New Sequence Variants in HLA Class II/III Region Associated with Susceptibility to Knee Osteoarthritis Identified by Genome-Wide Association Study
Source: PLoS One. 2010 Mar 18;5(3):e9723. doi: 10.1371/journal.pone.0009723 (PMC2841168; doi:10.1371/journal.pone.0009723)

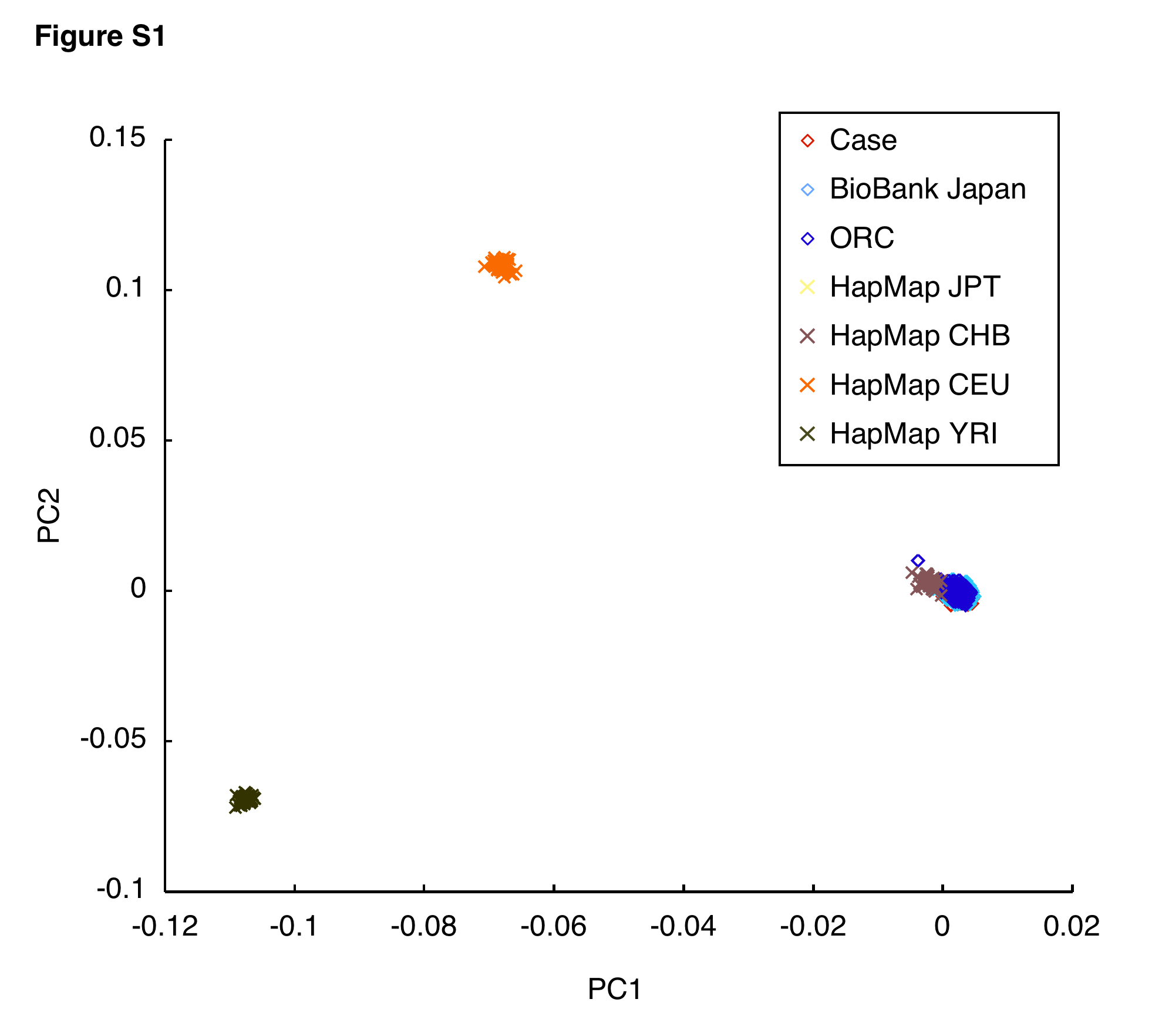

Supplement: Figure S1 — Principal component analysis of GWAS samples. Samples in the GWAS and in HapMap database are analyzed by a program of Smartpca [12], and plotted for the first (X axis) and the second (Y axis) principal component (PC), respectively. (0.16 MB TIF) [file pone.0009723.s001.tif]
